# Thiazole/Thiadiazole/Benzothiazole Based Thiazolidin-4-One Derivatives as Potential Inhibitors of Main Protease of SARS-CoV-2

**DOI:** 10.3390/molecules27072180

**Published:** 2022-03-28

**Authors:** Anthi Petrou, Panagiotis Zagaliotis, Nikoleta F. Theodoroula, George A. Mystridis, Ioannis S. Vizirianakis, Thomas J. Walsh, Athina Geronikaki

**Affiliations:** 1 School of Pharmacy, Faculty of Health Sciences, Aristotle University of Thessaloniki, 54124 Thessaloniki, Greece; anthi.petrou.thessaloniki1@gmail.com; 2 Division of Infectious Diseases, Weill Cornell Medicine, New York, NY 10065, USA; paz4002@med.cornell.edu; 3 Laboratory of Pharmacology, School of Pharmacy, Aristotle University of Thessaloniki, 54124 Thessaloniki, Greece; theodorn@pharm.auth.gr (N.F.T.); geormyst@pharm.auth.gr (G.A.M.); ivizir@pharm.auth.gr (I.S.V.); 4 Department of Life and Health Sciences, University of Nicosia, Nicosia CY-1700, Cyprus; 5 Center for Innovative Therapeutics and Diagnostics, Richmond, VA 23223, USA; thomaswalshmd@iitd.bio or

**Keywords:** SARS-CoV-2 main protease, inhibitors, COVID-19, docking studies, in vitro experiment

## Abstract

Since the time of its appearance until present, COVID-19 has spread worldwide, with over 71 million confirmed cases and over 1.6 million deaths reported by the World Health Organization (WHO). In addition to the fact that cases of COVID-19 are increasing worldwide, the Delta and Omicron variants have also made the situation more challenging. Herein, we report the evaluation of several thiazole/thiadiazole/benzothiazole based thiazolidinone derivatives which were chosen from 112 designed derivatives by docking as potential molecules to inhibit the main protease of SARS-CoV-2. The contained experimental data revealed that among the fifteen compounds chosen, five compounds (**k3**, **c1**, **n2**, **A2**, **A1**) showed inhibitory activity with IC_50_ within the range of 0.01–34.4 μΜ. By assessing the cellular effects of these molecules, we observed that they also had the capacity to affect the cellular viability of human normal MRC-5 cells, albeit with a degree of variation. More specifically, **k3** which is the most promising compound with the higher inhibitory capacity to SARS-CoV-2 protease (0.01 μΜ) affects in vitro cellular viability only by 57% at the concentration of 0.01 μM after 48 h in culture. Overall, these data provide evidence on the potential antiviral activity of these molecules to inhibit the main protease of SARS-CoV-2, a fact that sheds light on the chemical structure of the thiazole/thiadiazole/benzothiazole based thiazolidin-4-one derivatives as potential candidates for COVID-19 therapeutics.

## 1. Introduction

Structural Description of SARS-CoV-2 Main Protease

On 31 December 2019, several cases of pneumonia were reported in Wuhan [1], due to the novel coronavirus identified as Severe Acute Respiratory Syndrome Coronavirus 2 (SARS-CoV-2) which causes Coronavirus Disease 2019 (COVID-19) pandemic [2,3]. On 10 January 2020, the first genome of the new virus was deposited by Zhang et al. [3] on GenBank (MN908947) and other platforms.

Since the time of its appearance until present, COVID-19 has spread worldwide, with over 71 million confirmed cases and over 1.6 million deaths reported by the World Health Organization (WHO). In addition to the fact that cases of COVID-19 are increasing worldwide, the Delta and Omicron mutations have also made the situation more difficult. Τhe highest prevalence of cases is in America, followed by Europe and South-East Asia, while the lowest is in the Western Pacific.

This virus is very pathogenic and mostly affects respiratory tract cells, causing respiratory diseases that can develop into severe, even life-threatening, pathologies. The SARS-CoV-2 genome encodes nonstructural, structural, and accessory proteins [4], which play essential roles within the viral replicative cycle [5,6,7]. Spike protein, 3C-like protease (3CLpro), papain-like cysteine protease (PLpro), and RNA-dependent RNA polymerase (RdRp) are the main potential targets for antiviral therapeutics [8].

C376 is a pre-clinical dipeptide-based protease inhibitor, prodrug of GC373 another dipeptide-based protease inhibitor used against feline infectious peritonitis virus (FIPV), a strain of feline coronavirus (FCoV) [9,10]. Pedersen et al. [10] investigated the safety and efficacy of GC376 inhibitor by evaluation on client-owned cats with FIP (felic infectious peritonitis) and results were promising as regards the therapeutic efficacy. Fu et al. [9] reported that GC376 also inhibits SARS-CoV-2 in Vero cell by targeting the catalytically active sites of Mpro. Moreover, it possesses antiviral activity against SARS-CoV-2 with an EC50 value of 3.37 μM [11] and acts against MERS-CoV, which, together with SAR-CoV-2, is a coronavirus infecting human beings [12,13]. All these allow to consider GS376 inhibitor as a broad-spectrum antiviral drug, able to inhibit Mpro of a number of viruses, including many coronaviruses [12,13,14,15]. The fact that both GC373 and GC376 have high therapeutic index (>200) [14] can rapidly advance them to the stage, of the evaluation on human trials.

Despite all mentioned advantages of GC376, it was reported that upon treatment of FIP in cats, it created some side effects, such as subcutaneous fibrosis, transient stinging at the injection sites, hair loss, and abnormal eruption of permanent teeth in juvenile cats, suggesting more studies before use in clinical trials. Fu et al. [9] recommended a short period of using GC376 to treat COVID-19.

One of the key targets for the development of antiviral therapies against coronavirus is the main protease [16,17] which is involved in the cleavage of polyproteins, playing a crucial role in the replication cycle of the virus, making this enzyme an attractive target for potential anti-COVID drugs.

**Figure 1:**
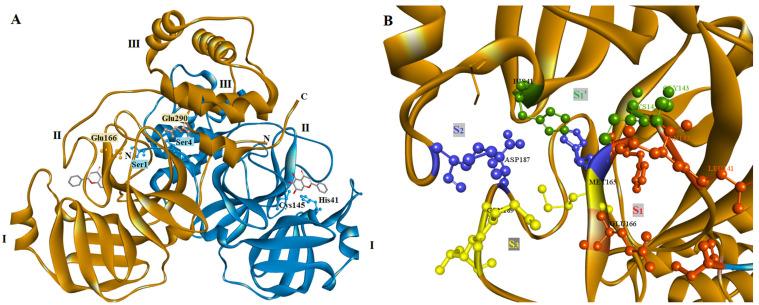
Structure of SARS-CoV-2 main protease. (**A**) Overview of the main protease homodimeric structure (PDBID 6M2N). Domains I, II and II are also shown. (**B**) Most important residues from the different subsites S3 (yellow), S2 (blue), S1 (red) and S1′ (green) of the binding site.

According to crystallographic studies, the main protease of SARS-CoV-2 is a homodimeric protease with two subunits (Figure 1A) [17]. Each subunit has a length of 306 residues and is formed by three domains, domain I include residues 8 to 100, domain II residues 101 to184 and domain III residues 199 to 306. The first two domains (I and II) have the same fold consisted of an antiparallel six-stranded β-barrel structure, while domain III is connected with domain II by a loop of residues 185–198 and is formed by five α-helices. Between domains I and II is located the substrate-binding site of the main protease while domain III is involved in the regulation of the main protease dimerization through a salt-bridge between the residue Arg4 from one protomer and Glu290 from the other [16,18]. This dimer formation is crucial for main protease activity because the N-terminal residue Ser1 of one protomer interacts with residue Glu166 of the other, forming the S1 subsite of the substrate-binding site of the enzyme [16] (Figure 1A).

The main protease binding site includes four subsites: S1, S1′, S3, and S4, with a catalytic dyad formed by residues His41 and Cys145. Most important residues of each subsite are His41, Gly143, Ser144, and Cys145 for S1′ subsite, Ser1 (from the other protomer), Phe140, Leu141, Asn142, His163 and Glu166 for S1 subsite, Met49, Tyr54, His164, Asp187 and Arg188 for S2 and Met165, Gln189, Thr190, and Gln192 for S3 subsite [16,19] (Figure 1B).

Recently, a number of main protease structures have been deposited into the Protein Data Bank, with the first of them being structure 6LU7 [17,20], giving the opportunity for in silico studies and structure-based design of the discovery of SARS-CoV-2 main protease inhibitors.

Molecular Docking studies are a powerful tool for rapid discovery of lead compounds for clinical use. Their big contribution is the significant reduction of cost and time, mainly for emerging diseases such as COVID-19 and the ability to speed up analyses of target interactions with drug candidates [21]. Using molecular docking we could model the interactions between a small molecule and a macromolecule such as a protein, as well as describe the behavior of small molecules in the binding site of proteins, and explain essential biochemical processes [22,23]. The docking method is comprised of the prediction of the ligand conformation, position, and orientation within each binding site and the calculation of the binding affinity.

**Figure 2:**
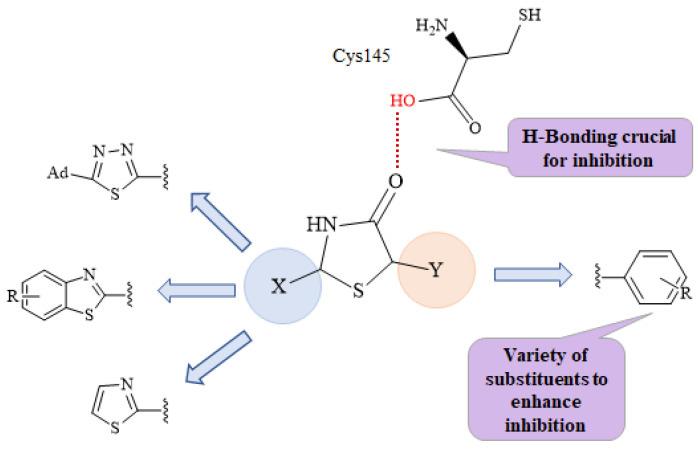
The design of novel main protease inhibitors. The H-bond is crucial to main protease binding (red).

The present research is a combination of traditional medicinal chemistry, structural biology, and computational chemistry. The new compounds will combine in their structure the minimum pharmacophores required to inhibit the main protease. In particular, they will be designed based on the following structural features and interactions provided to enhance their action:

It is known that the SARS-CoV-2 main protease cleaves its substrate after Gln, which follows Leu, and before a Ser or Ala or Gly amino acid (Leu-Gln ↓ Ser/Ala/Gly ↓ marks the cleavage site). Structure studies of the main protease enzyme with substrate analogues [17] showed that Gln is placed in the vicinity of residue Cys145, surrounded by the amino acids Asn142, Glu166, His163, and His172, while the amino acid Leu is in the vicinity and consists of the side chains of the amino acids His41, Met49, Tyr54, and Met165. The residues Cys145 and His41 act as a catalytic dyad consistent with the SARS chymotrypsin-like protease [24,25]. Therefore, a molecule that will interact strongly with these catalytic dyad residues may be the key to establishing a strong binding inhibition with this enzyme. In this direction, the presence of a thiazolidinone moiety seems to act as a mimetic of the Gln amino acid of the natural substrate. It could be placed at the S1 subsite in the active center of the enzyme, where Gln is naturally placed, between residues Glu166 and catalytic Cys145. Moreover, the oxygen atom of the CO group of thiazolidinone could form hydrogen bond interactions with the catalytic residue Cys145 (Figure 2).

The presence of a benzothiazole or thiazole moiety and aromatic rings, which, due to their hydrophobic nature, can form π-π interactions with the side chains of the residual amino acids His41, Gly143, Cys145, His163, Glu166, Met165, Gln189, and Gln192, enhancing the inhibition.

The presence of various substituents, and, especially halogens, are useful. Halogen substituents form electrostatic interactions, which are stronger that H-bonds, forming more stable complexes and consequently higher inhibition [26].

Taking all the above into account, after the design of the compounds, molecular docking studies will begin simultaneously with the calculation of the spectrum of biological activity of the compounds and the prediction of their pharmacokinetic profile in order to select for synthesis and in vitro studies those with the best probability of being potent inhibitors of the main protease enzyme.

## 2. Results

### 2.1. Chemistry

**Scheme 1:**
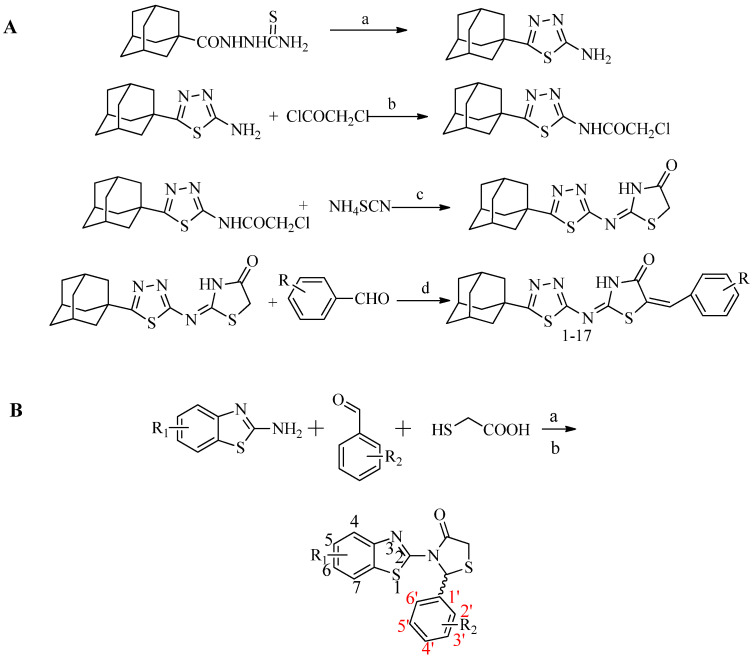
(**A**) Synthesis of 5-adamantyl-2-(1,3,4-thiadiazole)imino-5-arylidene-4-thiazolidinone. (**B**) Synthesis of 3-(2-hydroxyaryl)-1H-pyrazole derivatives.

Compounds were synthesized according to Scheme 1A,B, as described in our previous papers [27,28].

The structures of the newly synthesized compounds were confirmed by elemental analysis and spectroscopically (1H-NMR, ^13^C-NMR). In IR spectra stretching absorption bands at 1700 cm^−1^ (strong) of C=O, 1600 and 1540 cm^−1^ of -C–C- and 3200 of –OH were detected. In ^1^H-NMR spectra signal at 8.10–6.89 ppm, as well as at 4.35–4.10 ppm, are attributed to aromatic protons and protons of the position 2 of the thiazolidinone moiety, respectively. The rest of the protons appeared at the expected chemical shifts. In ^13^C-NMR, spectra peaks were observed for C=O group at δ 172–170 ppm, for C-2 of benzothiazole ring at δ 161–165 ppm, and for C-2 and C-5 of thiazolidinone moiety at 53–60 ppm and at 30–34 ppm, respectively.

### 2.2. Molecular Docking Prediction

It is known that the SARS-CoV-2 main protease cleaves its substrate after Gln, which follows Leu and before Ser or Ala or Gly amino acid (Leu-Gln ↓ Ser/Ala/Gly ↓ marks the cleavage site). Structure studies of the main protease enzyme with substrate analogues [10] showed that Gln is placed in vicinity to residue Cys145, surrounded by the amino acids Asn142, Glu166, His163, and His172, while the amino acid Leu in vicinity, which consists of the side chains of the amino acids His41, Met49, Tyr54, and Met165. The residues Cys145 and His41 act as a catalytic dyad consistent with the SARS chymotrypsin-like protease [24,25]. Therefore, a molecule that will interact strongly with these catalytic dyad residues may be the key to establishing a strong binding inhibition with this enzyme.

Taking these into account, we performed docking studies in a series of designed compounds in order to select those that will strongly bind to the SARS-CoV-2 main protease as possible inhibitors for further studies.

Docking analysis to a series of designed thiazolidinone compounds (Table 1) was performed using the SARS-CoV-2 main protease structure 6M2N. For the results, presented in Table 1, and from 112 compounds designed, we selected the 15 best for further studies as the most promising inhibitors with calculated free binding energy ranging from −8.63 to −10.78 kcal mol^−1^ (Table 1). Based on the literature and our previous experience, a value of free binding energy greater than −5.0 kcal mol^−1^ means that the compound is particularly inactive [29,30].

### 2.3. Biological Evaluation

Fifteen thiazolidinone derivatives were tested for their ability to inhibit the main protease of Sar-CoV-2. Three compounds are new, while the rest were synthesized and evaluated as antimicrobials previously [27,28]. The results are presented in Table 2.

The obtained results revealed that the best activity was shown by compound **k3** with IC_50_ at 0.010 μΜ, followed by compound **c1, n2** and **A2** with IC_50_ 4-736, 9.984, and 13.21 μΜ. Compound **m11** exhibited moderate activity, while the remaining compounds showed very low activity. It should be mentioned that the activity of compound **k3** is excided of that of the reference compound **GC376.**

According to the structure–activity relationship study, it is obvious that substituted 3-(benzo[d]thiazol-2-yl)-2-phenyl substituted thiazolidin-4-one derivatives (**c1, k3** and **n2**) are more potent than (2*E*,5*E*)-2-((5-((3r,5r,7r)-adamantan-1-yl)-1,3,4-thiadiazol- 2-yl)imino)-5-substituted benzylidenethiazolidin-4-ones (**A1, A2**). Thus, among the group of thiazolidin-4-one derivatives, the presence of 6-CN substituent on benzothiazole ring in combination with 2-Cl, 6-F substitution on a benzene ring (**k3**) is beneficial for the inhibitory activity replacement of 6-CN by 6-Cl and 2-Cl,6-F by 4-F substituents at benzothiazole and benzene rings, respectively, led to the second active compound **c1,** while 4-OH derivative (**c5**) was inactive. The presence of 4-Me, 6-adamantyl substitution on benzothiazole ring and 2,6-di-F at benzene ring (**n2**) was the third most active compound. Thus, the activity depends not only on the nature and position of substituents on a benzene ring, but also on a benzothiazole ring.

On the other hand, for 5-adamantan-1yl thiadiazole based thiazolidinones positively favorable for activity was the presence of 5-Ad and 2,6-di-F substitution on thiazole and benzene rings (**A2**), respectively. Replacement of 2,6-di-F substituent by 4-NO_2_ decreased activity by 2.6-fold. In this case, the activity depends on the nature and position of substituents at benzene ring.

### 2.4. Docking Studies

According to docking results, most of the tested compounds bind strongly to the SARS-CoV-2 main protease enzyme, forming π-π interactions with the side chains of the residual amino acids His41, Gly143, Cys145, Glu166, Met165, Gln189, and Gln192 enhancing the inhibition (Table 3).

**Figure 3:**
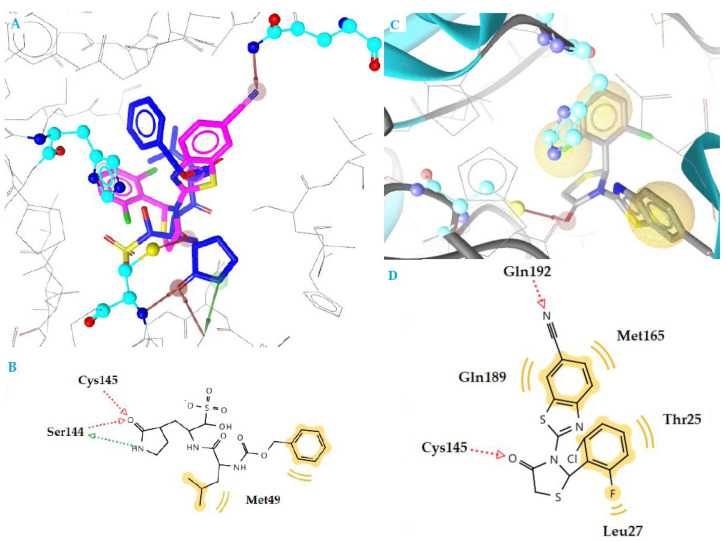
(**A**) Superposition of compound **k3** (magenta) and inhibitor **GC376** (blue) bound to SARS-CoV-2 main protease structure 6M2N, with specific residues labeled. (**B**) 2D interaction diagram of inhibitor **GC376** docking pose interactions with the key amino acids. (**C**) Docking pose of compound **k3** in SARS-CoV-2 main protease structure 6M2N. (**D**) 2D interaction diagram of compound **k3** docking pose interactions with the key amino acids. Red and green dotted arrows indicate H-bond and yellow spheres hydrophobic interactions.

The most active compound **k3** (calculated free binding energy −10.78 kcal mol^−1^) binds to the enzyme in a similar way as reference inhibitor **GC376** (Figure 3), with the thiazolidinone ring being placed at the S1 subsite in the active center of the enzyme, where Gln is naturally placed, between residues Glu166 and catalytic Cys145. The oxygen atom of CO group forms a hydrogen bond interaction with the residue Cys145 (distance 2.73 Å), while the nitrogen atom of CN substituent forms another hydrogen bond with Glu192 (distance 3.18 Å) (Figure 3C,D). Moreover, hydrophobic interactions are also formed between benzene moieties of compound and residues Thr25, Leu27, Met165, and Gln189, which contributes to complex stabilization.

**Figure 4:**
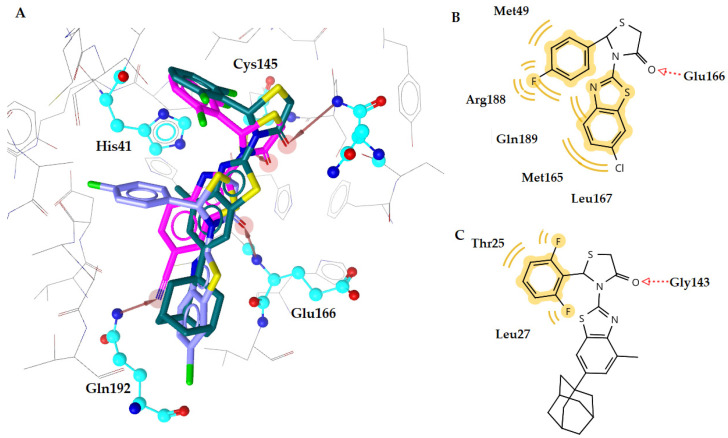
(**A**) Superposition of compounds **c1** (purple), **k3** (magenta) and **n2** (green) bound to SARS-CoV-2 main protease structure 6M2N, with specific residues labeled. Red arrows indicate H-bond interactions. (**B**) 2D interaction diagram of compound **c1** docking pose interactions with the key amino acids, (**C**) of compound **n2** in the active site of SARS-CoV-2 main protease structure 6M2N. Red dotted arrows indicate H-bond and yellow spheres hydrophobic interactions.

Compounds **c1** and **n2** adopt the same orientation inside the enzyme (Figure 4A). In compound **c1**, the oxygen atom of the C=O group of a thiazolidinone ring interacts with residue Glu166, forming a hydrogen bond, while in compound **n2,** the oxygen atom of C=O group is interacting with residue Gly143 (distance 3.18 Å and 3.57 Å, respectively). In addition, the benzene moieties of **c1** compound are involved in hydrophobic interactions with residues Met165, Lei167, Gln189, Arg188, and Met49 (Figure 4B,C). These interactions contribute further to stabilization of the complex **c1**-enzyme. On the other hand, compound **n2** interacts hydrophobically only throughout its benzene moiety with residues Thr25 and Leu27. This absence in stability may be the reason for the highest IC_50_ value of compound **n2** compared to compound **c1** (9.984 μΜ and 4.736 μΜ, respectively).

In general, the most active compounds **c1**, **k3,** and **n2** interact with most of the amino acids involved in complex stabilization of the natural substrate [16,17]. The thiazolidinone ring seems to act as a mimetic of the Gln amino acid of the natural substrate. Interestingly, the most promising compound **k3** interacts strongly with the catalytic dyad Cys145-His41 and the other two compounds interact with other crucial amino acids of the SARS-CoV-2 active site such as Glu166 and Met165, indicating a strong inhibition of the enzyme. These compounds showed excellent in silico results, characterized by their lower predicted free binding energy, which is reflected in their excellent in vitro anti-viral activity

### 2.5. Assessment of Cellular Viability

**Figure 5:**
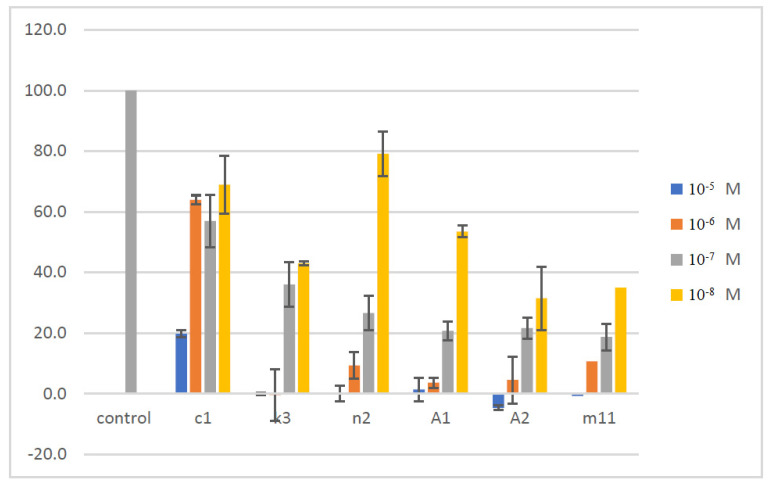
Effect of compounds on the cellular viability of human normal MRC-5 cells.

The synthesized compounds were assessed for their capacity to affect the cellular viability in the human normal MRC-5 cell line by applying the MTT cell viability assay. The effect of the compounds was evaluated by incubating each one separately in cell cultures for 48 h within the concentration range of 0.001 μM–10 μΜ (1 × 10^−8^ M–1 × 10^−5^ M). As shown in Figure 5, the cellular viability of MRC-5 was significantly inhibited by all the tested molecules in a dose-dependent manner compared to control untreated cultures. The most effective agents in reducing viability were **A2** and **m11,** followed by **A1**, **n2**, **k3** and **c1**. In particular, the compound **A2** caused inhibition of viability by 100% at 10 μM, 95.5% at 1 μM, 78.4% at 0.1 μM and 68.5% at 0.01 μM. Similarly, the effect of **m11** on viability was 100% at 10 μM, 89.4% at 1 μM, 81.3% at 0.1 μM and 65.0% at 0.01 μM. Further, for **A1** was 98.7% at 10 μM, 96.4% at 1 μM, 79.3% at 0.1 μM and 47.5% at 0.01 μM. The compound **n2** affected viability by 100% at 10 μM, 90.7% at 1 μM, 73.4.% at 0.1 μM and 20.8% at 0.01 μM. The compound **k3** affected viability by 100% at 10 μM, 100% at 1 μM, 64% at 0.1 μM and 57% at 0.01 μM. Finally, for the compound **c1** the inhibition of cellular viability was by 80.3% at 10 μM, 36.1% at 1 μM, 43,0% at 0.1 μM and by 31.0% at 0.01 μM. Overall, **k3** is the most promising compound since it exhibits the higher inhibitory capacity to SARS-COV-2 protease with IC50 = 0.01 μΜ and affects cellular viability only by 57% at this concentration after 48 h in culture.

## 3. Materials and Methods

### 3.1. Synthesis

*3-(5,6-dichlorobenzo[d]thiazol-2-yl)-2-(4-fluorophenyl)thiazolidin-4-one* (**a3**). Yield 81%. M p. 271–273 °C. IR (cm^−1^): 1748.38 (C=O). ^1^H NMR (500 MHz, Chloroform-*d*) δ 7.77 (d, *J* = 2.2 Hz, 1H), 7.60 (d, *J* = 8.7 Hz, 1H), 7.38–7.22 (m, 4H), 6.73 (s, 1H), 4.10 (dd, *J* = 16.6, 1.1 Hz, 1H), 3.86 (d, *J* = 16.6 Hz, 1H). ^13^C NMR (126 MHz Chloroform-*d*) δ 170.86, 156.10, 146.75, 138.89, 134.36, 133.31, 130.06, 129.10, 126.98, 126.92, 122.74, 120.85, 63.06, 63.04, 32.76.

*3-(6-(adamantan-1-yl)benzo[d]thiazol-2-yl)-2-(4-bromophenyl)thiazolidin-4-one* (**m11**). Yield 83%. M p. 275–276 °C. IR (cm^−1^): 1749.12 (C=O). ^1^H NMR (500 MHz, Chloroform-*d*) δ 7.74 (d, *J* = 1.9 Hz, 1H), 7.65 (d, *J* = 8.6 Hz, 1H), 7.42 (dd, *J* = 8.6, 1.9 Hz, 1H), 7.19 (tt, *J* = 8.5, 6.3 Hz, 1H), 7.11 (d, *J* = 1.3 Hz, 1H), 6.85 (t, *J* = 9.1 Hz, 2H), 4.27 (dd, *J* = 16.1, 1.3 Hz, 1H), 3.90 (dt, *J* = 16.1, 2.0 Hz, 1H), 2.11 (p, *J* = 3.1 Hz, 3H), 1.93 (d, *J* = 2.9 Hz, 6H), 1.84–1.70 (m, 7H), 1.32 (s, 1H). ^13^C NMR (126 MHz, Chloroform-*d*) δ 170.66, 155.38, 148.12, 146.11, 132.15, 130.08, 129.98, 123.54, 121.32, 117.08, 111.91, 111.72, 43.42, 36.70, 36.46, 33.83, 29.69, 28.93.

### 3.2. Molecular Docking

Molecular docking analysis was performed using the software Autodock 4.2 [31,32], as described in Supplementary Materials (S1. Molecular docking).

### 3.3. Inhibition of SARS-CoV-2 3CLpro Enzymatic Activity by Synthesized Compounds

The 3CLpro enzymatic assay was performed using SARS-CoV-2 specific 3CLpro assay kit, which was purchased from BPS Biosciences (Catalog #79955-1, San Diego, CA, USA). The assay was carried out following the manufacturer’s protocol. Briefly, 5 ng recombinant 3CLprotease-MBP tagged in 30 µL of assay buffer (with 1 mM dithiothreitol (DTT)) was pre-incubated with 10 µL of studied compounds, dissolved in DMSO (Sigma Aldrich, St. Louis, MO, USA), for 1 h. The enzymatic reaction was started by adding 10 µL fluorescent substrate. The assay samples had 50 µL final volume and 50 µM final concentrations of inhibitors and substrate in the reaction mixture. The incubation continued at room temperature for 12–17 h. The fluorescence intensity was measured by excitation/emission wavelength of 355/535 nm using PerkinElmer 2030 victor x multilabel plate reader. For IC_50_ calculation, samples were screened from 0.005 to 50 µM dose range. Wells with 5 ng of enzyme, 1% DMSO and substrate served as positive control with no enzyme inhibition, while wells with 100, 10 and 0,1 µM of inhibitor GC367 (BPS Biosciences) served as reference inhibitor. Wells without enzyme, 1% DMSO and substrate served as blank.

GraphPad Prism 9 program with non-linear regression (curve fit) was used to calculate the IC_50_ values of tested compounds.

The IC_50_ value of reference inhibitor GC376, provided by BPS Biosciences was found to be 0.439 μM, in accordance with the manufacture’s protocol value, validating the in vitro assay.

### 3.4. Evaluation of Cellular Viability by MTT Assay

The normal human lung fibroblast MRC-5 cell line is stored and used in our laboratory in a routine manner (passage < 40). MRC-5 cells were grown at 37 °C in humidified atmosphere containing 5% *v*/*v* CO2 by applying DMEM medium supplemented with 10% *v*/*v* FBS, 1% PS penicillin-streptomycin. The compounds tested were dissolved in DMSO and stored in 4 °C. For the assessment of cellular viability, the cells were seeded at an initial concentration of 5 × 10^4^ cells/mL in 96-well plates. After at least 3 h following cell attachment to the plate, each compound was added in the cultures at four different concentrations: 1 × 10^−5^ M (10 μΜ), 1 × 10^−6^ M (1 μΜ), 1 × 10^−7^ M (0.1 μΜ) and 1 × 10^−8^ M (0.01 μΜ). Note that the concentration of DMSO in culture was ≤0.2% *v*/*v*, in which no detectable effect on cell proliferation is observed. To evaluate the capacity of each compound to affect viability, the cells were allowed to grow for an additional 48 h. At this point, 10 μL of MTT (Trevigen, Gaithersburg, MD, USA) was warmed to 37 °C, homogenized, and added (100 μL) to each well. After 1 h, the water-soluble yellow MTT was converted into the water-insoluble purple formazan by the metabolically active cells, and the formazan was further dissolved by the addition of 50 μL of DMSO solvent. The 96-well plate was covered with aluminum foil and shaken for 15 min in a plate shaker. The resultant product was quantified by spectrophotometry using a plate reader at 660 nm. Some wells were left cell-free to act as controls to test the absorption capacity of the compounds and/or the medium (i.e., as blank controls). The results were then expressed as percentages compared to control untreated cultures. For each individual concentration, at least 3 independent cell cultures were used to allow statistical analysis. The data were analyzed using one-way analysis of variance (ANOVA) and statistical significance was set at *p* < 0.05 [33].

## 4. Conclusions

Fifteen compounds out of 112 designed were selected based on molecular docking for the synthesis and evaluation of SARS-CoV-2 main protease inhibitory activity. Five out of the fifteen tested compounds exhibited inhibitory action with IC_50_ ranging from 0.010–13.21 μΜ. One of them showed better activity (IC_50_ 0.010 μΜ) than **GC376** (IC_50_ 0.439 μΜ) under experimental conditions.

According to docking results, five active compounds strongly bind SARS-CoV-2 main protease enzyme forming π-π interactions with the side chains of the residual amino acids His41, Gly143, Cys145, Glu166, Met165, Gln189, and Gln192, enhancing the inhibition.

In general, the most active compounds **c1**, **k3,** and **n2** interact with most of the amino acids involved in complex stabilization of the natural substrate. A thiazolidinone ring seems to act as a mimetic of the Gln amino acid of the natural substrate. These compounds showed excellent in silico results, characterized by their lower predicted free binding energy, which is reflected in their excellent in vitro anti-viral activity. However, these five active compounds exhibit the capacity to affect the viability of human normal MRC-5 cells by exhibiting a dose-dependent effect and variability to their response. Importantly, **k3** is shown to be the most promising compound with the higher inhibitory capacity to SARS-COV2 protease (0.01 μΜ) that only affects cellular viability by 57% at this concentration after 48 h in culture. Further structural modifications may be able to yield compounds that retain potent activity against the main protease while also minimizing in vitro cytotoxicity.

Overall, these studies provide new insights on the potential pharmacological exploitation of thiazole/thiadiazole/benzothiazole based thiazolidinone derivatives as potential SARS-COV2 protease inhibitors, as well as promising drug candidates for COVID-19 therapy.

## Figures and Tables

**Table 1:**
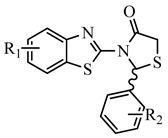
Estimated free binding energies of designed compounds to the crystal structure of SARS-CoV-2 main protease (PDB code: 6M2N).

Nο	R_1_	R_2_	Est. Free Binding Energy (kcal/mol)) S-(−)	Est. Free Binding Energy (kcal/mol) R-(+)	Νο	R_1_	R_2_	Est. Free Binding Energy (kcal/mol) S-(−)	Est. Free Binding Energy (kcal/mol) R-(+)
**a1**	7-Cl	2,6-di-F	−8.57	−9.27	**i7**	6-Br	2,3-di-Cl	−6.19	−7.04
**a2**	7-Cl	2-F, 6-Cl	−2.79	−3.54	**i8**	6-Br	2,4-di-Cl	−5.23	−5.67
**a3**	4,5-di-Cl	4-F	−9.16	−9.58	**k1**	6-CN	4-NO_2_	−4.60	−4.11
**b1**	6-F	4-F	−8.57	−8.93	**k2**	6-CN	2,6-di-F	−5.38	−5.61
**b2**	6-F	4-NO_2_	−6.37	−6.88	**k3**	6-CN	2-F, 6-Cl	−10.44	−10.78
**b3**	6-F	4-Cl	−6.91	−7.12	**k4**	6-CN	2,6-di-Cl	−4.37	−5.60
**b4**	6-F	4-OCH_3_	−5.23	−6.17	**k5**	6-CN	4-F	−6.35	−6.99
**b5**	6-F	4-OH	−5.64	−5.34	**k6**	6-CN	2,4-di-Cl	−6.14	−7.01
**b6**	6-F	4-Br	−6.92	−5.80	**l1**	6-CF_3_	2,6-di-Cl	−5.33	−5.10
**b7**	6-F	2,3-di-Cl	−6.93	−5.86	**l2**	6-CF_3_	2,6-di-F	−4.03	−3.96
**b8**	6-F	2,4-di-Cl	−5.61	−5.72	**l3**	6-CF_3_	2-F, 6-Cl	−6.17	−7.00
**c1**	6-Cl	4-F	−10.25	−10.70	**l4**	6-CF_3_	4-Br	−5.29	−6.33
**c2**	6-Cl	4-NO_2_	−3.49	−4.11	**l5**	6-CF_3_	2,3-di-Cl	−4.24	−4.58
**c3**	6-Cl	4-Cl	−3.02	−4.34	**l6**	6-CF_3_	2,4-di-Cl	−5.20	−5.87
**c4**	6-Cl	4-OCH_3_	−4.71	−5.06	**m1**	6-Ad	2,6-di-Cl	−6.33	−6.32
**c5**	6-Cl	4-OH	−9.72	−9.85	**m2**	6-Ad	2-F, 6-Cl	−9.57	−10.16
**c6**	6-Cl	4-Br	−2.19	−3.21	**m3**	6-Ad	2,6-di-F	−5.44	−5.72
**c7**	6-Cl	2,3-di-Cl	−4.25	−6.14	**m4**	6-Ad	2,3-di-Cl	−6.38	−6.10
**c8**	6-Cl	2,4-di-Cl	−3.75	−4.66	**m5**	6-Ad	2,4-di-Cl	−5.13	−6.70
**d1**	4-Cl	4-F	−8.51	−8.63	**m6**	6-Ad	4-F	−2.16	−3.88
**d2**	4-Cl	4-NO_2_	−6.13	−6.45	**m7**	6-Ad	4-NO_2_	−1.03	−2.67
**d3**	4-Cl	4-Cl	−5.29	−6.74	**m8**	6-Ad	4-Cl	−5.30	−3.46
**d4**	4-Cl	4-OCH_3_	−7.22	−7.50	**m9**	6-Ad	4-OCH_3_	−6.90	−7.10
**d5**	4-Cl	4-OH	−6.97	−7.82	**m10**	6-Ad	4-OH	−2.88	−3.67
**d6**	4-Cl	4-Br	−4.63	−5.19	**m11**	6-Ad	4-Br	−8.91	−9.03
**d7**	4-Cl	2,3-di-Cl	−2.94	−3.68	**n1**	4-CH_3_, 6-Ad	2,6-di-Cl	−7.34	−7.66
**d8**	4-Cl	2,4-di-Cl	−5.26	−6.28	**n2**	4-CH_3_, 6-Ad	2,6-di-F	−10.10	−10.12
**e1**	4-OCH_3_	4-F	−5.37	−6.27	**n3**	4-CH_3_, 6-Ad	2-F, 6-Cl	−7.10	−7.91
**e2**	4-OCH_3_	4-NO_2_	−8.62	−8.90	**n4**	4-CH_3_, 6-Ad	2,3-di-Cl	−6.52	−7.04
**e3**	4-OCH_3_	4-Cl	−6.49	7.13	**n5**	4-CH_3_, 6-Ad	2,4-di-Cl	−8.33	−8.42
**e4**	4-OCH_3_	4-OCH_3_	−5.41	−6.82	**n6**	4-CH_3_, 6-Ad	4-F	−6.27	−6.44
**e5**	4-OCH_3_	4-OH	−5.34	−5.33	**n7**	4-CH_3_, 6-Ad	4-NO_2_	−5.12	−5.49
**e6**	4-OCH_3_	4-Br	−6.27	−6.64	**n8**	4-CH_3_, 6-Ad	4-Cl	−4.67	−6.74
**f1**	6-OCH_3_	4-F	−5.22	−6.39	**o1**	5,6-di-CH_3_	4-F	−5.14	−6.30
**f2**	6-OCH_3_	4-NO_2_	−5.10	−6.82	**o2**	5,6-di-CH_3_	4-NO_2_	−2.83	−2.61
**f3**	6-OCH_3_	4-Cl	−3.78	−4.09	**o3**	5,6-di-CH_3_	4-Cl	−3.66	−4.72
**f4**	6-OCH_3_	4-OCH_3_	−3.38	−3.56	**o4**	5,6-di-CH_3_	4-OCH_3_	−3.28	−4.56
**h1**	6-OCF_3_	2,6-di-Cl	−5.13	−6.72	**q1**	4-CH_3_	4-F	−5.67	−5.91
**h2**	6-OCF_3_	2,6-di-F	−6.23	−4.97	**q2**	4-CH_3_	4-NO_2_	−7.05	−7.40
**h3**	6-OCF_3_	2-F, 6-Cl	−1.37	−4.06	**q3**	4-CH_3_	4-Cl	−6.55	−6.81
**h4**	6-OCF_3_	2,3-di-Cl	−5.62	−4.09	**q4**	4-CH_3_	4-OCH_3_	−5.00	−5.37
**h5**	6-OCF_3_	2,4-di-Cl	−2.88	−1.86	**q5**	4-CH_3_	4-OH	−4.10	−6.89
**h6**	6-OCF_3_	4-F	−9.05	−9.17	**q6**	4-CH_3_	4-Br	−2.56	−2.88
**h7**	6-OCF_3_	4-NO_2_	−5.42	−5.69	**q7**	4-CH_3_	2,3-di-Cl	−3.65	−4.78
**h8**	6-OCF_3_	4-Cl	−8.63	−8.80	**q8**	4-CH_3_	2,4-di-Cl	−2.31	−3.79
**h9**	6-OCF_3_	4-OCH_3_	−4.37	−4.66	**r1**	6-CH_3_	4-F	−5.30	−6.71
6M2N Initial inhibitor *			−10.45		**GC376**			−10.35	

* 6M2N Initial inhibitor: 5,6,7-trihydroxy-2-phenyl-4*H*-chromen-4-one.

**Table 2:**
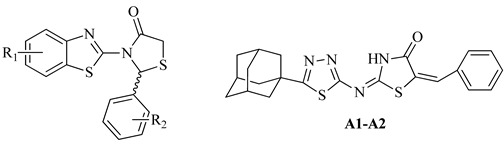
% Inhibition at 50μΜ and IC_50_ values of tested compounds for SARS-CoV-2 3CL protease.

No	R1	R2	% Inhibition at 50 μM	IC_50_ (μΜ)
**a3**	4,5-di-Cl	4-F	0	>50
**a1**	7-Cl	2,6-di-F	0	>50
**b1**	6-F	4-F	0	>50
**c1**	6-Cl	4-F	88.03	4.736
**c5**	6-Cl	4-OH	7.87	>50
**d1**	4-Cl	4-F	6.72	>50
**e2**	4-OCH_3_	4-NO_2_	31.30	>50
**h6**	6-OCF_3_	4-F	25.18	>50
**h8**	6-OCF_3_	4-Cl	3.04	>50
**k3**	6-CN	2-F, 6-Cl	100	**0.010**
**n2**	4-Me, 6-Ad	2-F, 6-Cl	100	9.984
**m2**	6-Ad	2-F, 6-Cl	2.79	>50
**m11**	6-Ad	4-Br	45.00	>50
**A1**	6-Ad	4-NO_2_	94.11	34.4
**A2**	6-Ad	2,6-di-F	91.34	13.21
**GC376**			100 at 100 μM	0.439

**Table 3:** Estimated free binding energies of tested compounds in the crystal structure of SARS-CoV-2 main protease (PDB code: 6M2N).

No	Est. Free Binding Energy (kcal/mol)	I-H	Residues Involved in Hydrogen Bonds	Hydrophobic Interactions
**a1**	−9.27	1	Arg188	Asn142, Gly143, His164
**a3**	−9.58	1	Asn142	Met49, Glu166, Leu167
**b1**	−8.93	1	Gln192	His164, Asp187, Gln189
**c1**	−10.70	1	**Glu166**	Met49, Met165, Leu167, Arg188, Gln189
**c5**	−9.85	1	**Glu166**	Leu27, His164, Gln189
**d1**	−8.63	1	Gln192	Thr25, Leu27, Gln189
**e2**	−8.90	1	Gln192	Leu27, Asp187
**h6**	−9.17	1	**Glu166**	Leu27, Tyr54, Arg188
**h8**	−8.80	1	Gln192	Thr25, Met165
**k3**	−10.78	2	**Cys145**, Gln192	Thr25, Leu27, Met165, Gln189
**n2**	−10.12	1	Gly143	Thr25, Leu27
**m2**	−10.16	1	**Glu166**	Leu27, Met165
**m11**	−9.03	1	**Glu166**	Leu27, Gln189
6M2N Initial inhibitor *	−10.45	2	Gly143, **Glu166**	Leu27, Tyr54, Asn42, His164, Gln189, Arg188, Asp187
**GC376**	−11.35	3	Ser144, **Cys145**	Met49

* 6M2N Initial inhibitor: 5,6,7-trihydroxy-2-phenyl-4H-chromen-4-one.

## Data Availability

Not applicable.

## Supplementary Materials

The following supporting information can be downloaded at: https://www.mdpi.com/article/10.3390/molecules27072180/s1, S1. Molecular docking: Figure S1: Docking of the initial ligand 5,6,7-trihydroxy-2-phenyl-4H-chromen-4-one to the SARS-CoV-2 main protease structure 6M2N. The docked ligand is in green and the initial ligand; Table S1: Smiles of all compounds; ^1^H-NMR and ^13^C-NMR of newly synthesized compounds.

## References

[1] WHO (2020). Novel Coronavirus e China. https://www.who.int/csr/don/12-january-2020-novel-coronavirus-china/en/.

[2] Zhu N., Zhang D., Wang W. (2020). A novel coronavirus from patients with pneumonia in China, 2019. N. Engl. J. Med..

[3] Huang C., Wang Y., Li X. (2020). Clinical features of patients infected with 2019 novel coronavirus in Wuhan, China. Lancet.

[4] Sun J., He W.T., Wang L., Lai A., Ji X., Zhai X., Li G., Suchard M.A., Tian J., Zhou J. (2020). COVID-19: Epidemiology, Evolution, and Cross-Disciplinary Perspectives. Trends Mol. Med..

[5] Du L., He Y., Zhou Y., Liu S., Zheng B.J., Jiang S. (2009). The spike protein of SARS-CoV—A target for vaccine and therapeutic development. Nat. Rev. Microbiol..

[6] Walls A.C., Park Y.J., Tortorici M.A., Wall A., McGuire A.T., Veesler D. (2020). Structure, Function, and Antigenicity of the SARS-CoV-2 Spike Glycoprotein. Cell.

[7] Lu R., Zhao X., Li J., Niu P., Yang B., Wu H., Wang W., Song H., Huang B., Zhu N. (2020). Genomic characterisation and epidemiology of 2019 novel coronavirus: Implications for virus origins and receptor binding. Lancet.

[8] Ghanbari R., Teimoori A., Sadeghi A., Mohamadkhani A., Rezasoltani S., Asadi E., Jouyban A., Sumner S.C. (2020). Existing antiviral options against SARS-CoV-2 replication in COVID-19 patients. Future Microbiol..

[9] Fu L., Ye F., Feng Y., Yu F., Wang Q., Wu Y., Zhao C., Sun H., Huang B., Niu P. (2020). Both Boceprevir and GC376 efficaciously inhibit SARS-CoV-2 by targeting its main protease. Nat. Commun..

[10] Pedersen N.C., Kim Y., Liu H., Galasiti Kankanamalage A.C., Eckstrand C., Groutas W.C., Bannasch M., Meadows J.M., Chang K.O. (2018). Efficacy of a 3C-like protease inhibitor in treating various forms of acquired feline infectious peritonitis. J. Feline Med. Surg..

[11] Ma C., Sacco M.D., Hurst B. (2020). Boceprevir, GC-376, and calpain inhibitors II, XII inhibit SARS-CoV-2 viral replication by targeting the viral main protease. Cell Res..

[12] Rathnayake A.D., Zheng J., Kim Y., Perera K.D., Mackin S., Meyerholz D.K., Kashipathy M.M., Battaile K.P., Lovell S., Perlman S. (2020). 3C-like protease inhibitors block coronavirus replication in vitro and improve survival in MERS-CoV-infected mice. Sci. Transl. Med..

[13] Wang Y.C., Yang W.H., Yang C.S., Hou M.H., Tsai C.L., Chou Y.Z., Hung M.C., Chen Y. (2020). Structural basis of SARS-CoV-2 main protease inhibition by a broad-spectrum anti-coronaviral drug. Am. J. Cancer Res..

[14] Vuong W., Khan M.B., Fischer C. (2020). Feline coronavirus drug inhibits the main protease of SARS-CoV-2 and blocks virus replication. Nat. Commun..

[15] Perera K.D., Galasiti Kankanamalage A.C., Rathnayake A.D., Honeyfield A., Groutas W., Chang K.O., Kim Y. (2018). Protease inhibitors broadly effective against feline, ferret and mink coronaviruses. Antivir. Res..

[16] Zhang L., Lin D., Sun X., Curth U., Drosten C., Sauerhering L., Becker S., Rox K., Hilgenfeld R. (2020). Crystal structure of SARS-CoV-2 main protease provides a basis for design of improved α-ketoamide inhibitors. Science.

[17] Jin Z., Du X., Xu Y., Deng Y., Liu M., Zhao Y., Zhang B., Li X., Zhang L., Peng C. (2020). Structure of Mpro from COVID-19 virus and discovery of its inhibitors. Nature.

[18] Shi J., Song J. (2006). The catalysis of the SARS 3C-like protease is under extensive regulation by its extra domain. FEBS J..

[19] Tang B., He F., Liu D., Fang M., Wu Z., Xu D. (2020). AI-aided design of novel targeted covalent inhibitors against SARS-CoV-2. bioRxiv.

[20] Yin S., Huang M., Li D.M., Tang N. (2021). Difference of coagulation features between severe pneumonia induced by SARS-CoV2 and non-SARS-CoV2. J. Thromb. Thrombolysis.

[21] Gimeno A., Ojeda-Montes M., Tomás-Hernández S., Cereto-Massagué A., Beltrán-Debón R., Mulero M., Pujadas G., Garcia-Vallvé S. (2019). The Light and Dark Sides of Virtual Screening: What Is There to Know?. Int. J. Mol. Sci..

[22] McConkey B.J., Sobolev V., Edelman M. (2002). The performance of current methods in ligand-protein docking. Curr. Sci..

[23] Meng X.Y., Zhang H.X., Mezei M., Cui M. (2011). Molecular docking: A powerful approach for structure-based drug discovery. Curr. Comput. Aided Drug Des..

[24] Qamar M.T., Alqahtani S.M., Alamri M.A. (2020). Structural basis of SARS-CoV-2 3CLpro and anti-COVID-19 drug discovery from medicinal plants. J. Pharm. Sci..

[25] Yang H.T., Yang M.J., Ding Y. (2003). The crystal structures of severe acute respiratory syndrome virus main protease and its complex with an inhibitor. Proc. Natl. Acad. Sci. USA.

[26] Metrangolo P., Resnati G. (2001). Halogen bonding: A paradigm in supramolecular chemistry. Chemistry.

[27] Fesatidou M., Zagaliotis P., Camoutsis C., Petrou A., Ciric A., Sokovic M. (2018). 5-Adamantan thiadiazole-based thiazolidinones as antimicrobial agents. Design, synthesis, molecular docking and evaluation. Bioorg. Med. Chem..

[28] Petrou A., Eleftheriou P., Geronikaki A., Akrivou M.G., Vizirianakis I. (2019). Novel thiazolidin-4-ones as potential non-nucleoside inhibitors of HIV-1 reverse transcriptase. Molecules.

[29] Eleftheriou P., Petrou A., Geronikaki A., Liaras K., Dirnali S., Anna M. (2015). Prediction of enzyme inhibition and mode of inhibitory action based on calculation of distances between hydrogen bond donor/acceptor groups of the molecule and docking analysis: An application on the discovery of novel effective PTP1B inhibitors. SAR QSAR Environ. Res..

[30] Eleftheriou P., Amanatidou D., Petrou A., Geronikaki A. (2020). In silico evaluation of the effectivity of approved protease inhibitors against the main protease of the novel SARS-CoV-2 virus. Molecules.

[31] Morris G.M., Huey R., Lindstrom W., Sanner M.F., Belew R.K., Goodsell D.S., Olson A.J. (2009). Autodock4 and AutoDockTools4: Automated docking with selective receptor flexiblity. J. Comput. Chem..

[32] Ganou C.A., Eleftheriou P.T., Theodosis-Nobelos P., Geronikaki A.A., Lialiaris T., Rekka E.A. (2018). Docking analysis targeted to the whole enzyme: An application to the prediction of inhibition of PTP1b by thiomorpholine and thiazolyl derivatives. SAR QSAR Environ. Res..

[33] Akrivou M.G., Demertzidou V.P., Theodoroula N.F., Chatzopoulou F.M., Kyritsis K.A., Grigoriadis N., Zografos A.L., Vizirianakis I.S. (2018). Uncovering the pharmacological response of novel sesquiterpene derivatives that differentially alter gene expression and modulate the cell cycle in cancer cells. Int. J. Oncol..

