# Supplementary material for: Thiazole/Thiadiazole/Benzothiazole Based Thiazolidin-4-One Derivatives as Potential Inhibitors of Main Protease of SARS-CoV-2
Source: Molecules. 2022 Mar 28;27(7):2180. doi: 10.3390/molecules27072180 (PMC9000570; doi:10.3390/molecules27072180)
Supplement: Supplementary file 1 [file molecules-27-02180-s001.zip › molecules-1638214-supplementary.pdf]

Thiazole/thiadiazole/benzothiazole based thiazolidin-4-one derivatives as potential inhibitors of main protease of SARS-CoV-2

Anthi Petrou [1], Panagiotis Zagaliotis [2,3], Nikoleta F. Theodoroula [3], George A. Mystridis [3], Ioannis S. Vizirianakis [3,,4], Thomas J. Walsh [5] and Athina Geronikaki [1,*]

[1] School of Pharmacy, Faculty of Health Sciences, Aristotle University of Thessaloniki, 54124 Thessaloniki, Greece; anthi.petrou.thessaloniki1@gmail.com
[2] Division of Infectious Diseases, Weill Cornell Medicine, 10065, New York, NY, USA; paz4002@med.cornell.edu
[3] Laboratory of Pharmacology,School of Pharmacy, Aristotle University of Thessaloniki, 54124 Thessaloniki, Greece; geormyst@pharm.auth.gr (G.A.M.); ivizir@pharm.auth.gr (I.S.V.); theodorn@pharm.auth.gr (N.F.T.)
[4] Department of Life and Health Sciences, University of Nicosia, CY-1700 Nicosia, Cyprus (I.S.V.)
[5] Institute for Innovative Therapeutics and Diagnostics, Richmond, VA, USA; Thomaswalshmd@iitd.bio; thomaswalshmd@gmail.com
* Correspondence: Correspondence: geronik@oharm.auth.gr

S1. Molecular docking

Molecular docking analysis was performed using the software Autodock 4.2 [31]. The Lamarckian genetic algorithm was applied for minimization using default parameters. The number of docking runs was 100 that were set to terminate after a maximum of 2,500,000 energy evaluations, all rotatable torsions were released and the population size was set to 150. A translational step of 0.2 Å, and quaternion and torsion steps of 5 were applied during the search. After docking, the 100 solutions were clustered into groups with RMS lower than 1.0 E. The clusters were ranked by the lowest energy representative of each cluster. In order to describe the ligand-binding pocket interactions, the top ranked binding mode was found by Autodock in complex with the binding pocket of the enzyme. The resulting poses and potential interactions were visualized in Discovery Studio 2017 R2 Client and LigandScout program.

The structure of SARS-CoV-2 main protease enzyme in a complex with the inhibitor 5,6,7-trihydroxy-2-phenyl-4H-chromen-4-one was chosen from the Protein Data Bank (PDB ID: 6M2N) [17]. This structure was preferred for the in-silico studies because the PDB file includes both subunits of the enzyme and it can enable a probable interaction of the second subunit in binding of the compounds. It is important to highlight that residue Glu166 of the one subunit interacts with Ser1 of the other subunit of the protein and usually participates in complex stabilization.

For docking analysis, the docking center was kept at coordinates x = −61.73, y = −35.18, z = 23.26 and the target box was set at x = 20, y = 20 and z = 20 to surround the binding site of the inhibitor (active site of the enzyme). Furthermore, to ensure that the tested compounds will bind only to the active site of the enzyme and not to other sides, the docking was repeated, extending the docking box to cover the whole enzyme [32].

For the evaluation of the docking method, the initial inhibitor 5,6,7-trihydroxy-2- phenyl-4H-chromen-4-one was removed and docked back to the enzyme and was compared with its initial position at the complex with RMSD value 0.38Å (Figure S1).

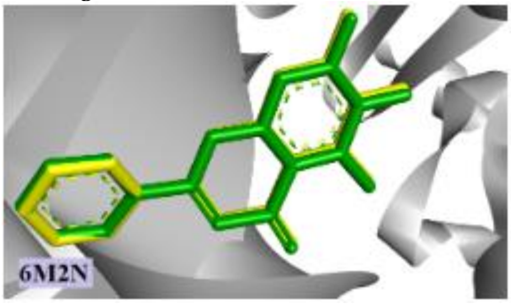

Figure S1. Docking of the initial ligand 5,6,7-trihydroxy-2-phenyl-4H-chromen-4-one to the SARS- CoV-2 main protease structure 6M2N. The docked ligand is in green and the initial ligand.

Table S1. Smiles of compounds.

No	Smiles	No	Smiles
**a1**	O=C1N([C@H](C2=C(F)C=CC=C2F)SC1)C3=NC4=C(S3)C(Cl)=CC=C4	**i7**	O=C1N([C@H](C2=CC=CC(Cl)=C2Cl)SC1)C3=NC4=C(S3)C=C(Br)C=C4
**a2**	O=C1N([C@H](C2=C(Cl)C=CC=C2F)SC1)C3=NC4=C(S3)C(Cl)=CC=C4	**i8**	O=C1N([C@H](C2=CC=C(Cl)C=C2Cl)SC1)C3=NC4=C(S3)C=C(Br)C=C4
**a3**	O=C1N([C@H](C2=CC=C(F)C=C2)SC1)C3=NC4=C(S3)C=C(Cl)C(Cl)=C4	**k1**	O=C1N([C@H](C2=CC=C([N+]([O-])=O)C=C2)SC1)C3=NC4=C(S3)C=C(C#N)C=C4
**b1**	O=C1N([C@H](C2=CC=C(F)C=C2)SC1)C3=NC4=C(S3)C=C(F)C=C4	**k2**	O=C1N([C@H](C2=C(F)C=CC=C2F)SC1)C3=NC4=C(S3)C=C(C#N)C=C4
**b2**	O=C1N([C@H](C2=CC=C([N+]([O-])=O)C=C2)SC1)C3=NC4=C(S3)C=C(F)C=C4	**k3**	O=C1N([C@H](C2=C(F)C=CC=C2Cl)SC1)C3=NC4=C(S3)C=C(C#N)C=C4
**b3**	O=C1N([C@H](C2=CC=C(Cl)C=C2)SC1)C3=NC4=C(S3)C=C(F)C=C4	**k4**	O=C1N([C@H](C2=C(Cl)C=CC=C2Cl)SC1)C3=NC4=C(S3)C=C(C#N)C=C4
**b4**	O=C1N([C@H](C2=CC=C(OC)C=C2)SC1)C3=NC4=C(S3)C=C(F)C=C4	**k5**	O=C1N([C@H](C2=CC=C(F)C=C2)SC1)C3=NC4=C(S3)C=C(C#N)C=C4
**b5**	O=C1N([C@H](C2=CC=C(O)C=C2)SC1)C3=NC4=C(S3)C=C(F)C=C4	**k6**	O=C1N([C@H](C2=C(Cl)C=C(Cl)C=C2)SC1)C3=NC4=C(S3)C=C(C#N)C=C4
**b6**	O=C1N([C@H](C2=CC=C(Br)C=C2)SC1)C3=NC4=C(S3)C=C(F)C=C4	**l1**	O=C1N([C@H](C2=C(Cl)C=CC=C2Cl)SC1)C3=NC4=C(S3)C=C(C(F)(F)F)C=C4
**b7**	O=C1N([C@H](C2=C(Cl)C=CC=C2Cl)SC1)C3=NC4=C(S3)C=C(F)C=C4	**l2**	O=C1N([C@H](C2=C(F)C=CC=C2F)SC1)C3=NC4=C(S3)C=C(C(F)(F)F)C=C4
**b8**	O=C1N([C@H](C2=C(Cl)C=C(Cl)C=C2)SC1)C3=NC4=C(S3)C=C(F)C=C4	**l3**	O=C1N([C@H](C2=C(Cl)C=CC=C2F)SC1)C3=NC4=C(S3)C=C(C(F)(F)F)C=C4
**c1**	O=C1N([C@H](C2=CC=C(F)C=C2)SC1)C3=NC4=C(S3)C=C(Cl)C=C4	**l4**	O=C1N([C@H](C2=CC=C(Br)C=C2)SC1)C3=NC4=C(S3)C=C(C(F)(F)F)C=C4
**c2**	O=C1N([C@H](C2=CC=C([N+]([O-])=O)C=C2)SC1)C3=NC4=C(S3)C=C(Cl)C=C4	**l5**	O=C1N([C@H](C2=C(Cl)C(Cl)=CC=C2)SC1)C3=NC4=C(S3)C=C(C(F)(F)F)C=C4
**c3**	O=C1N([C@H](C2=CC=C(Cl)C=C2)SC1)C3=NC4=C(S3)C=C(Cl)C=C4	**l6**	O=C1N([C@H](C2=C(Cl)C=C(Cl)C=C2)SC1)C3=NC4=C(S3)C=C(C(F)(F)F)C=C4
**c4**	O=C1N([C@H](C2=CC=C(OC)C=C2)SC1)C3=NC4=C(S3)C=C(Cl)C=C4	**m1**	O=C1N([C@H](C2=C(Cl)C=CC=C2Cl)SC1)C3=NC4=C(S3)C=C(C5(C6)C[C@@H](C[C@@H]6C7)C[C@@H]7C5)C=C4
**c5**	O=C1N([C@H](C2=CC=C(O)C=C2)SC1)C3=NC4=C(S3)C=C(Cl)C=C4	**m2**	O=C1N([C@H](C2=C(Cl)C=CC=C2F)SC1)C3=NC4=C(S3)C=C(C5(C6)C[C@@H](C[C@@H]6C7)C[C@@H]7C5)C=C4
**c6**	O=C1N([C@H](C2=CC=C(Br)C=C2)SC1)C3=NC4=C(S3)C=C(Cl)C=C4	**m3**	O=C1N([C@H](C2=C(F)C=CC=C2F)SC1)C3=NC4=C(S3)C=C(C5(C6)C[C@@H](C[C@@H]6C7)C[C@@H]7C5)C=C4
**c7**	O=C1N([C@H](C2=CC=CC(Cl)=C2Cl)SC1)C3=NC4=C(S3)C=C(Cl)C=C4	**m4**	O=C1N([C@H](C2=C(Cl)C(Cl)=CC=C2)SC1)C3=NC4=C(S3)C=C(C5(C6)C[C@@H](C[C@@H]6C7)C[C@@H]7C5)C=C4
**c8**	O=C1N([C@H](C2=CC=C(Cl)C=C2Cl)SC1)C3=NC4=C(S3)C=C(Cl)C=C4	**m5**	O=C1N([C@H](C2=C(Cl)C=C(Cl)C=C2)SC1)C3=NC4=C(S3)C=C(C5(C6)C[C@@H](C[C@@H]6C7)C[C@@H]7C5)C=C4
**d1**	O=C1N([C@H](C2=CC=C(F)C=C2)SC1)C3=NC4=C(S3)C=CC=C4Cl	**m6**	O=C1N([C@H](C2=CC=C(F)C=C2)SC1)C3=NC4=C(S3)C=C(C5(C6)C[C@@H](C[C@@H]6C7)C[C@@H]7C5)C=C4
**d2**	O=C1N([C@H](C2=CC=C([N+]([O-])=O)C=C2)SC1)C3=NC4=C(S3)C=CC=C4Cl	**m7**	O=C1N([C@H](C2=CC=C([N+]([O-])=O)C=C2)SC1)C3=NC4=C(S3)C=C(C5(C6)C[C@@H](C[C@@H]6C7)C[C@@H]7C5)C=C4
**d3**	O=C1N([C@H](C2=CC=C(Cl)C=C2)SC1)C3=NC4=C(S3)C=CC=C4Cl	**m8**	O=C1N([C@H](C2=CC=C(Cl)C=C2)SC1)C3=NC4=C(S3)C=C(C5(C6)C[C@@H](C[C@@H]6C7)C[C@@H]7C5)C=C4
**d4**	O=C1N([C@H](C2=CC=C(OC)C=C2)SC1)C3=NC4=C(S3)C=CC=C4Cl	**m9**	O=C1N([C@H](C2=CC=C(O[CH])C=C2)SC1)C3=NC4=C(S3)C=C(C5(C6)C[C@@H](C[C@@H]6C7)C[C@@

			H]7C5)C=C4
**d5**	O=C1N([C@H](C2=CC=C(O)C=C2)SC1)C3=NC4=C(S3)C=CC=C4Cl	**m10**	O=C1N([C@H](C2=CC=C(O)C=C2)SC1)C3=NC4=C(S3)C=C(C5(C6)C[C@@H](C[C@@H]6C7)C[C@@H]7C5)C=C4
**d6**	O=C1N([C@H](C2=CC=C([B])C=C2)SC1)C3=NC4=C(S3)C=CC=C4Cl	**m11**	O=C1N([C@H](C2=CC=C(Br)C=C2)SC1)C3=NC4=C(S3)C=C(C5(C6)C[C@@H](C[C@@H]6C7)C[C@@H]7C5)C=C4
**d7**	O=C1N([C@H](C2=C(Cl)C(Cl)=CC=C2)SC1)C3=NC4=C(S3)C=CC=C4Cl	**n1**	O=C1N([C@H](C2=C(Cl)C=CC=C2Cl)SC1)C3=NC4=C(S3)C=C(C5(C6)C[C@@H](C[C@@H]6C7)C[C@@H]7C5)C=C4C
**d8**	O=C1N([C@H](C2=C(Cl)C=C(Cl)C=C2)SC1)C3=NC4=C(S3)C=CC=C4Cl	**n2**	O=C1N([C@H](C2=C(F)C=CC=C2F)SC1)C3=NC4=C(S3)C=C(C5(C6)C[C@@H](C[C@@H]6C7)C[C@@H]7C5)C=C4
**e1**	O=C1N([C@H](C2=CC=C(F)C=C2)SC1)C3=NC4=C(S3)C=CC=C4OC	**n3**	O=C1N([C@H](C2=C(Cl)C=CC=C2F)SC1)C3=NC4=C(S3)C=C(C5(C6)C[C@@H](C[C@@H]6C7)C[C@@H]7C5)C=C4C
**e2**	O=C1N([C@H](C2=CC=C(N=O)C=C2)SC1)C3=NC4=C(S3)C=CC=C4OC	**n4**	O=C1N([C@H](C2=C(Cl)C(Cl)=CC=C2)SC1)C3=NC4=C(S3)C=C(C5(C6)C[C@@H](C[C@@H]6C7)C[C@@H]7C5)C=C4C
**e3**	O=C1N([C@H](C2=CC=C(Cl)C=C2)SC1)C3=NC4=C(S3)C=CC=C4OC	**n5**	O=C1N([C@H](C2=C(Cl)C=C(Cl)C=C2)SC1)C3=NC4=C(S3)C=C(C5(C6)C[C@@H](C[C@@H]6C7)C[C@@H]7C5)C=C4C
**e4**	O=C1N([C@H](C2=CC=C(O[CH])C=C2)SC1)C3=NC4=C(S3)C=CC=C4OC	**n6**	O=C1N([C@H](C2=CC=C(F)C=C2)SC1)C3=NC4=C(S3)C=C(C5(C6)C[C@@H](C[C@@H]6C7)C[C@@H]7C5)C=C4C
**e5**	O=C1N([C@H](C2=CC=C(O)C=C2)SC1)C3=NC4=C(S3)C=CC=C4OC	**n7**	O=C1N([C@H](C2=CC=C([N+]([O-])=O)C=C2)SC1)C3=NC4=C(S3)C=C(C5(C6)C[C@@H](C[C@@H]6C7)C[C@@H]7C5)C=C4C
**e6**	O=C1N([C@H](C2=CC=C(Br)C=C2)SC1)C3=NC4=C(S3)C=CC=C4OC	**n8**	O=C1N([C@H](C2=CC=C(Cl)C=C2)SC1)C3=NC4=C(S3)C=C(C5(C6)C[C@@H](C[C@@H]6C7)C[C@@H]7C5)C=C4C
**f1**	O=C1N([C@H](C2=CC=C(F)C=C2)SC1)C3=NC4=C(S3)C=C(OC)C=C4	**o1**	O=C1N([C@H](C2=CC=C(F)C=C2)SC1)C3=NC4=C(S3)C=C(C)C(C)=C4
**f2**	O=C1N([C@H](C2=CC=C([N+]([O-])=O)C=C2)SC1)C3=NC4=C(S3)C=C(OC)C=C4	**o2**	O=C1N([C@H](C2=CC=C([N+]([O-])=O)C=C2)SC1)C3=NC4=C(S3)C=C(C)C(C)=C4
**f3**	O=C1N([C@H](C2=CC=C(Cl)C=C2)SC1)C3=NC4=C(S3)C=C(OC)C=C4	**o3**	O=C1N([C@H](C2=CC=C(Cl)C=C2)SC1)C3=NC4=C(S3)C=C(C)C(C)=C4
**f4**	O=C1N([C@H](C2=CC=C(OC)C=C2)SC1)C3=NC4=C(S3)C=C(OC)C=C4	**o4**	O=C1N([C@H](C2=CC=C(OC)C=C2)SC1)C3=NC4=C(S3)C=C(C)C(C)=C4
**h1**	O=C1N([C@H](C2=C(Cl)C=CC=C2Cl)SC1)C3=NC4=C(S3)C=C(OC(F)(F)F)C=C4	**q1**	O=C1N([C@H](C2=CC=C(F)C=C2)SC1)C3=NC4=C(S3)C=CC=C4C
**h2**	O=C1N([C@H](C2=C(F)C=CC=C2F)SC1)C3=NC4=C(S3)C=C(OC(F)(F)F)C=C4	**q2**	O=C1N([C@H](C2=CC=C([N+]([O-])=O)C=C2)SC1)C3=NC4=C(S3)C=CC=C4C
**h3**	O=C1N(C2=NC3=CC=C(OC(F)(F)F)C=C3S2)[C@@H](SC1)C4=C(Cl)C=CC=C4F	**q3**	O=C1N([C@H](C2=CC=C(Cl)C=C2)SC1)C3=NC4=C(S3)C=CC=C4C
**h4**	O=C1N([C@H](C2=CC=CC(Cl)=C2Cl)SC1)C3=NC4=C(S3)C=C(OC(F)(F)F)C=C4	**q4**	O=C1N([C@H](C2=CC=C(OC)C=C2)SC1)C3=NC4=C(S3)C=CC=C4C
**h5**	O=C1N([C@H](C2=CC=C(Cl)C=C2Cl)SC1)C3=NC4=C(S3)C=C(OC(F)(F)F)C=C4	**q5**	O=C1N([C@H](C2=CC=C(O)C=C2)SC1)C3=NC4=C(S3)C=CC=C4C
**h6**	O=C1N([C@H](C2=CC=C(F)C=C2)SC1)C3=NC4=C(S3)C=C(OC(F)(F)F)C=C4	**q6**	O=C1N([C@H](C2=CC=C(Br)C=C2)SC1)C3=NC4=C(S3)C=CC=C4C
**h7**	O=C1N([C@H](C2=CC=C([N+]([O-])=O)C=C2)SC1)C3=NC4=C(S3)C=C(OC(F)(F)F)C=C4	**q7**	O=C1N([C@H](C2=C(Cl)C(Cl)=CC=C2)SC1)C3=NC4=C(S3)C=CC=C4C
**h8**	O=C1N([C@H](C2=CC=C(Cl)C=C2)SC1)C3=NC4=C(S3)C=C(OC(F)(F)F)C=C4	**q8**	O=C1N([C@H](C2=C(Cl)C=C(Cl)C=C2)SC1)C3=NC4=C(S3)C=CC=C4C
**h9**	O=C1N([C@H](C2=CC=C(OC)C=C2)SC1)C3=NC4=C(	**r1**	O=C1N([C@H](C2=CC=C(F)C=C2)SC1)C3=NC4=C(S

S3)C=C(OC(F)(F)F)C=C4	3)C=C(C)C=C4

Spectra for newly synthesized compounds

Compound a3

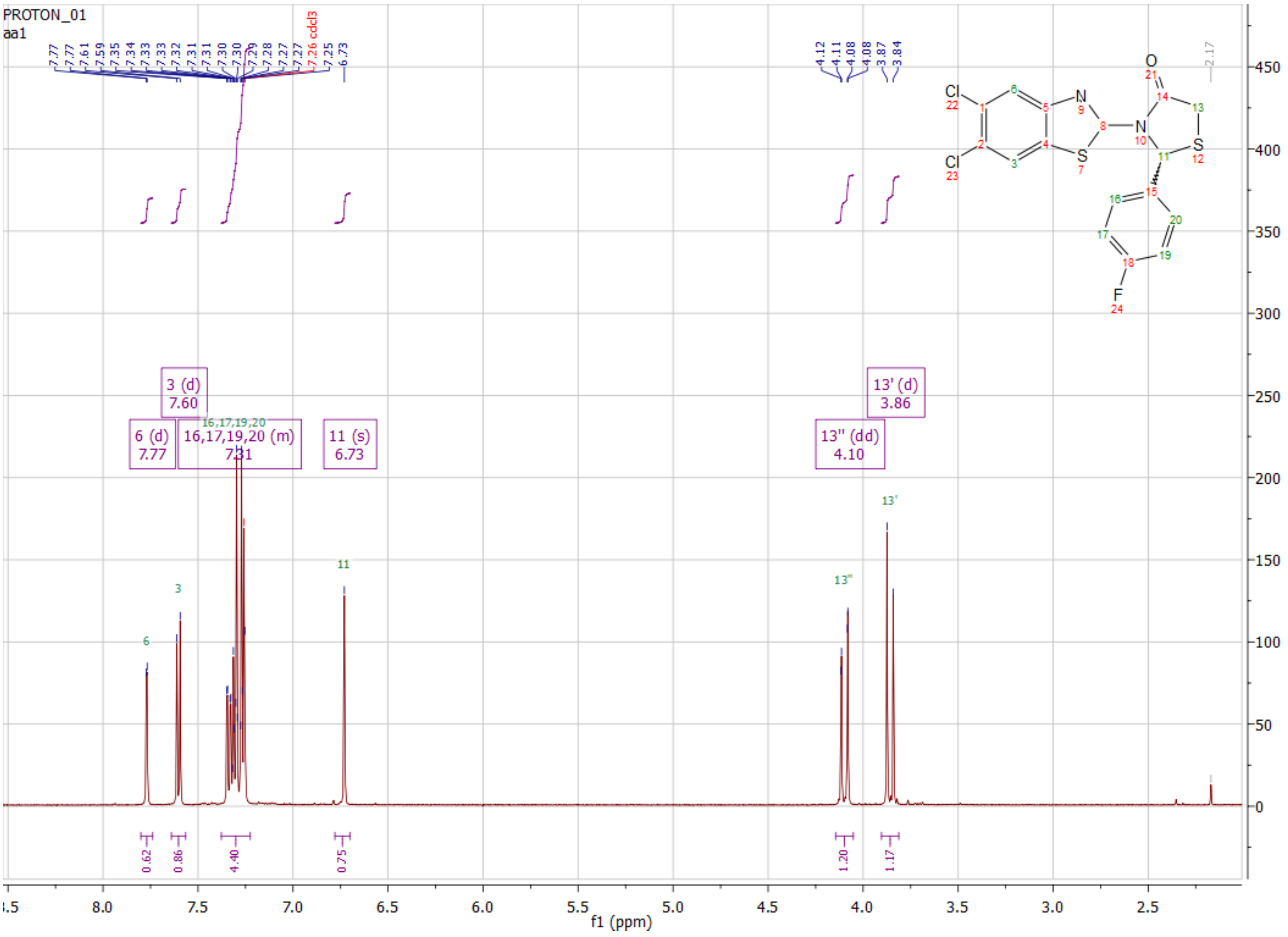

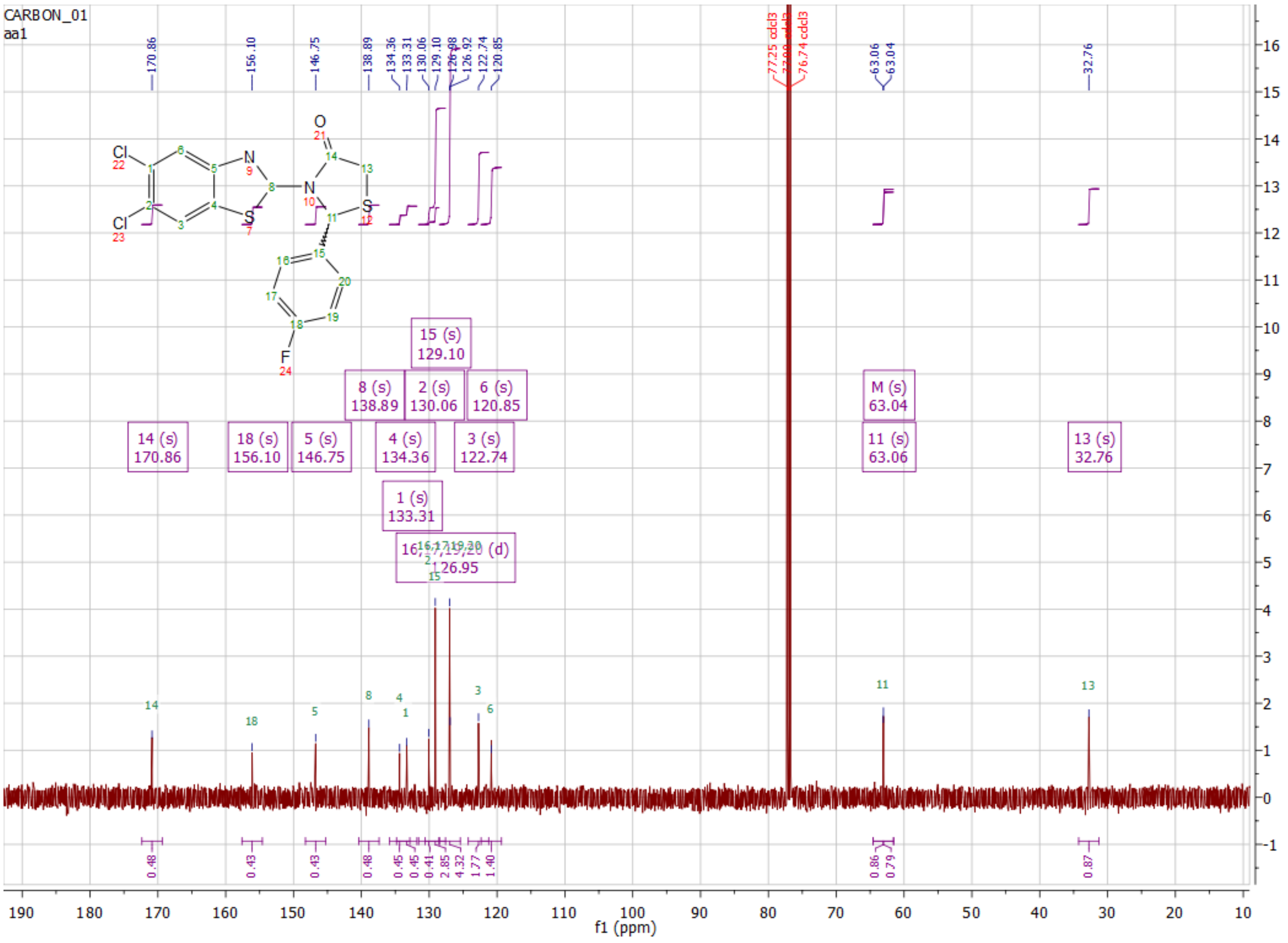

Compound m11

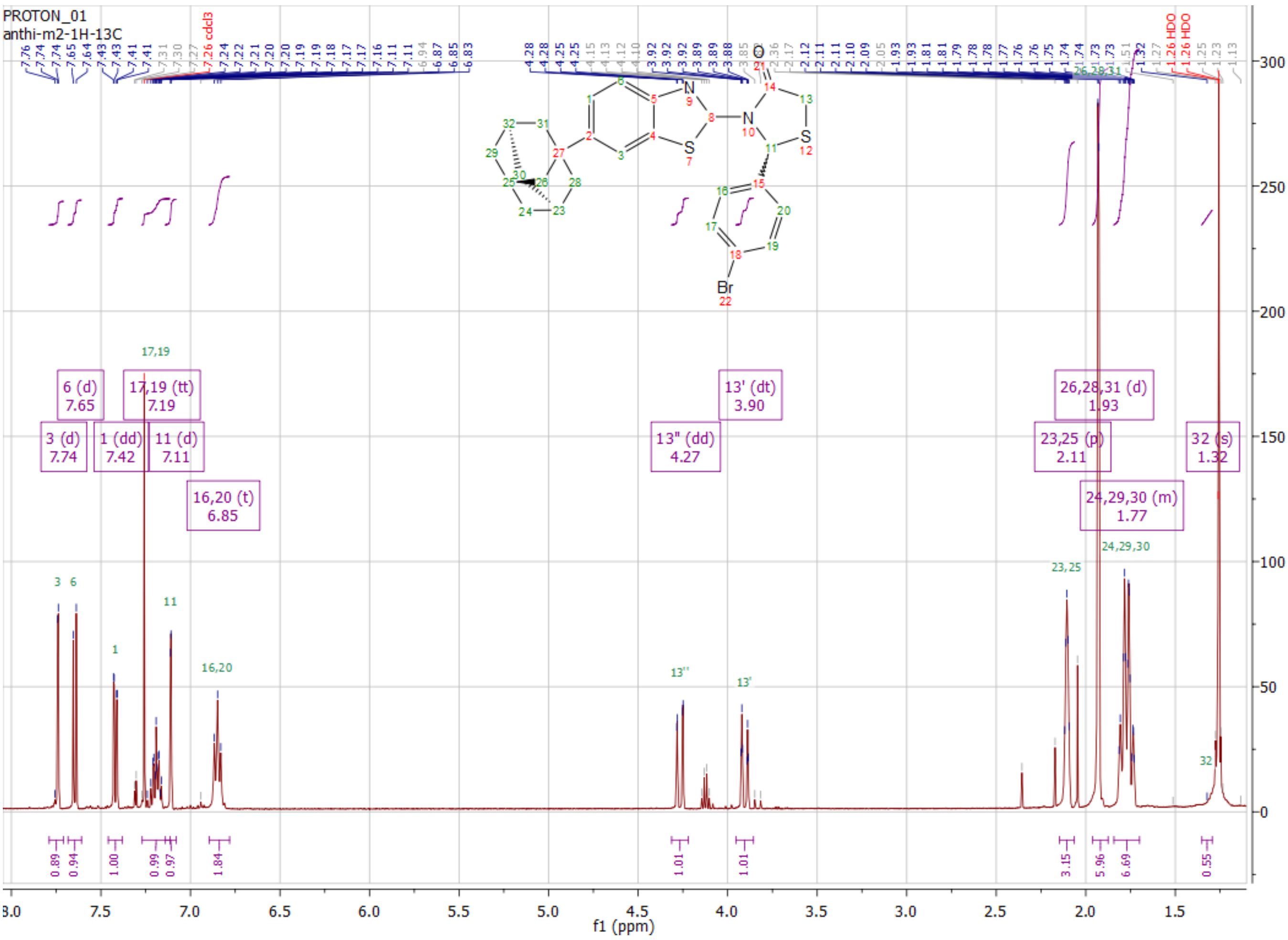

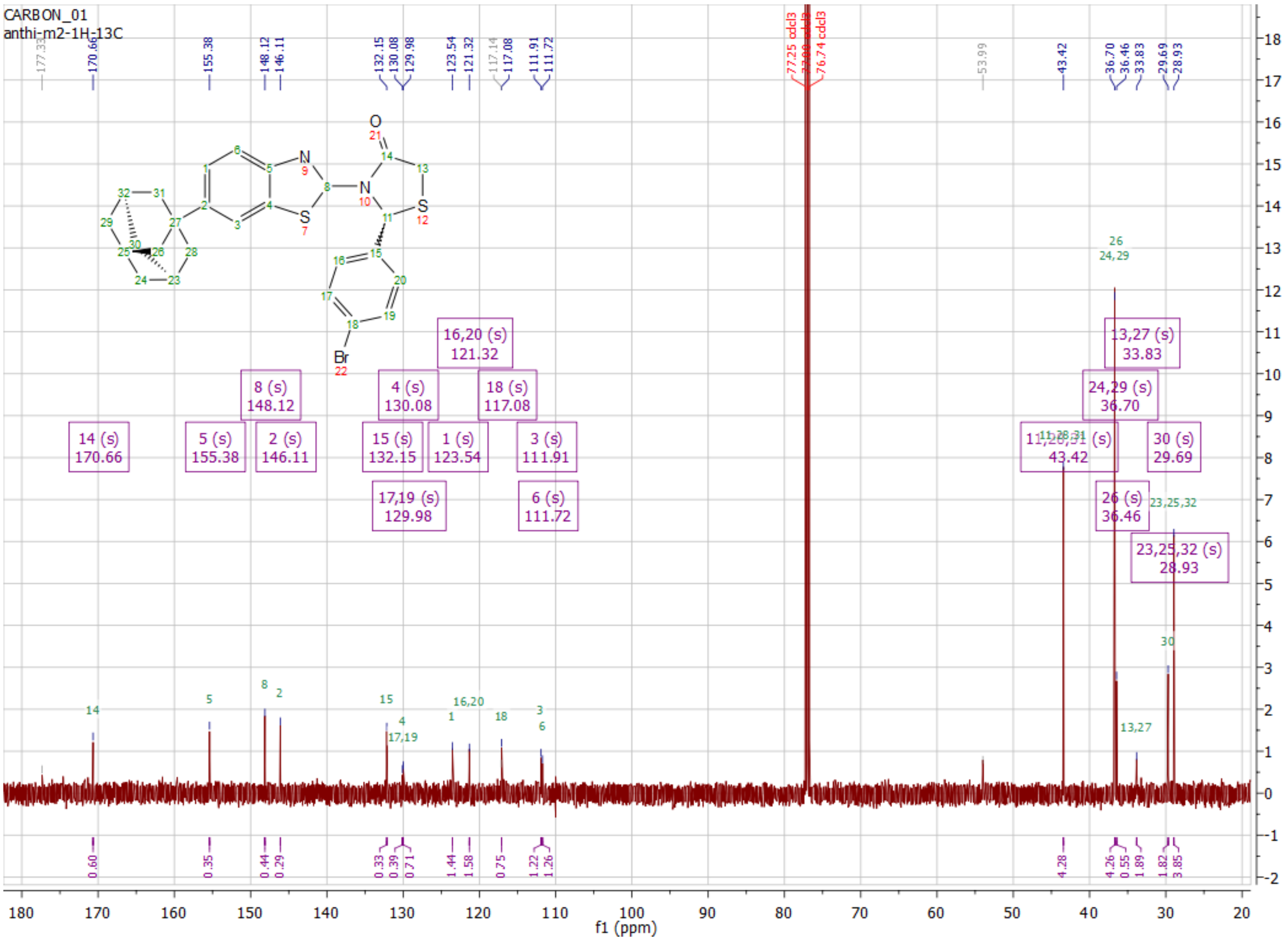
CARBON_01
anthi-m2-1H-13C
14 (s) 170.66
5 (s) 155.38
8 (s) 148.12
2 (s) 146.11
15 (s) 132.15
4 (s) 130.08
17,19 (s) 129.98
1 (s) 123.54
16,20 (s) 121.32
18 (s) 117.08
3 (s) 111.91
6 (s) 111.72
43.42
24,29 (s) 36.70
26 (s) 36.46
13,27 (s) 33.83
30 (s) 29.69
23,25,32 (s) 28.93
f1 (ppm)
